# Mapping sulphadoxine-pyrimethamine-resistant *Plasmodium falciparum* malaria in infected humans and in parasite populations in Africa

**DOI:** 10.1038/s41598-017-06708-9

**Published:** 2017-08-07

**Authors:** Lucy C. Okell, Jamie T. Griffin, Cally Roper

**Affiliations:** 1 MRC Centre for Outbreak Analysis and Modelling, Department of Infectious Disease Epidemiology, Imperial College London, London, UK; 2 School of Mathematical Sciences, Queen Mary University of London, London, UK; 3 Department of Pathogen Molecular Biology, Faculty of Infectious and Tropical Diseases, London School of Hygiene & Tropical Medicine, London, UK

## Abstract

Intermittent preventive treatment (IPT) with sulphadoxine-pyrimethamine in vulnerable populations reduces malaria morbidity in Africa, but resistance mutations in the parasite *dhps* gene (combined with *dhfr* mutations) threaten its efficacy. We update a systematic review to map the prevalence of K540E and A581G mutations in 294 surveys of infected humans across Africa from 2004-present. Interpreting these data is complicated by multiclonal infections in humans, especially in high transmission areas. We extend statistical methods to estimate the frequency, i.e. the proportion of resistant clones in the parasite population at each location, and so standardise for varying transmission levels. Both K540E and A581G mutations increased in prevalence and frequency in 60% of areas after 2008, highlighting the need for ongoing surveillance. Resistance measures within countries were similar within 300 km, suggesting an appropriate spatial scale for surveillance. Spread of the mutations tended to accelerate once their prevalence exceeded 10% (prior to fixation). Frequencies of resistance in parasite populations are the same or lower than prevalence in humans, so more areas would be classified as likely to benefit from IPT if similar frequency thresholds were applied. We propose that the use of resistance frequencies as well as prevalence measures for policy decisions should be evaluated.

## Introduction

Intermittent preventive treatment in infants (IPTi) and pregnant women (IPTp) with sulphadoxine–pyrimethamine (SP) is recommended by the World Health Organization (WHO) in areas of moderate-to-high malaria transmission in sub-Saharan Africa but wide regional variations in drug resistance critically influence the success of this intervention. In East Africa, parasites have a higher threshold of SP tolerance than those found in West Africa. Mutations in dihydrofolate reductase (*Pfdhfr* gene) confer resistance to pyrimethamine while mutations in the dihydropteroate synthetase (*Pfdhps* gene) confer resistance to sulphadoxine. The association between these mutations and SP efficacy is complex^1^ and is affected by factors such as individuals’ immunity to malaria^2^, but the following associations have been found. In East Africa a triple mutant *dhfr* allele containing N51I, C59R, and S108N mutations combined with a double mutant *dhps* (A437G + K540E) (together known as the quintuple mutant) is associated with clinical and parasitological SP treatment failure^3–6^, and reduces the prophylactic period provided by SP^6^. However, protection by IPT with SP in pregnant women against low birth weight outcomes is sustained even in areas with high levels of the quintuple mutant^6–9^, and infants benefit from IPT in areas with intermediate (~50% prevalence), though not high (>90% prevalence) levels of the quintuple mutant^10, 11^. In certain East African foci, resistance has intensified because parasites have acquired an additional *dhps* A581G mutation. One such area reported 86% clinical failure^12^ and loss of protective efficacy of IPTi^13^ and IPTp^13, 14^, and a meta-analysis concluded that IPTp efficacy was reduced when the prevalence of the 581G mutation was over 10%^2, 14, 15^. Hence the surveillance and reporting of 540E and 581G has a central role in IPTp-SP policy decisions.

**Figure 1:**
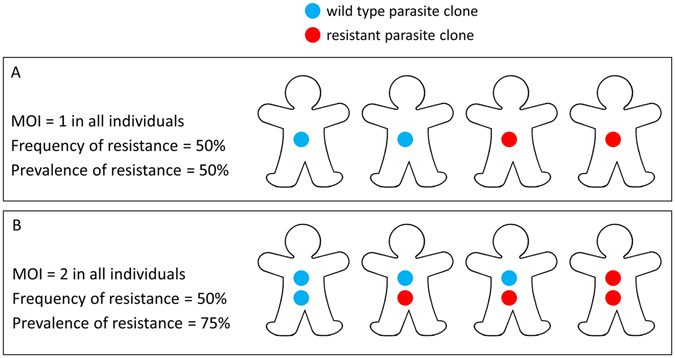
The difference between the prevalence of resistance in the infected human population (% of individuals carrying at least one resistant parasite clone) and the frequency of resistance in the parasite population (% of parasite clones which are resistant). If each individual carries only one parasite clone (a multiplicity of infection (MOI) of 1) (**A**), then the frequency and prevalence of resistance are the same. However in most areas, particularly areas with high malaria transmission intensity, individuals carry multiple parasite clones, so that the frequency and prevalence of resistance are often not the same. For example, if everyone in an area carries 2 parasite clones (**B**), and the frequency of resistance in the parasite population is 50%, then the prevalence of resistance in humans would be 75% if clones are distributed randomly. The prevalence of resistance in humans is almost always higher than the frequency of resistance in the parasite population due to multi-clonal infections. In reality, individuals in a malaria-endemic area carry different numbers of parasite clones (which we allow for in the analyses presented in the Methods and Results sections).

The WHO recommends IPTi with SP only where the prevalence of 540E among infected individuals is under 50%^10^, and the WHO draft recommendations on IPTp recommend considering discontinuation of IPTp when the prevalence of 581G is over 10% and 540E is over 95%^16^. The prevalence of resistance mutations is defined as the proportion of infected humans who are carrying at least one mutant parasite clone. The prevalence is readily quantified from PCR analysis of the haploid parasite blood stage in fingerprick blood samples from infected humans. However, interpreting these data is complicated by individuals infected with multiple parasite clones, especially in areas of high transmission^17, 18^. The frequency of resistance in the parasite population, defined as the proportion of parasite clones which have a resistance marker, is usually not the same as the prevalence of resistance in infected humans (Fig. 1). For example, in a situation where the frequency of resistance in parasites is 50%, the prevalence of resistance in humans will also be 50% if every infected individual carries only one parasite clone, but would be 75% if everyone carried 2 clones (assuming random distribution of clones) (Fig. 1). In mixed infections containing wild type and resistant parasites with more than 2 clones (a multiplicity of infection (MOI) >2), frequencies cannot be directly measured using standard PCR techniques, because these simply detect the presence or absence of resistant and wild type parasites, not their abundance^19^. For example in mixed infections with an MOI of 3, there could be 1 resistant clone and 2 wild type, or 2 resistant clones and 1 wild type. The unit of analysis for resistance frequency used throughout our study is a parasite clone, in line with previous analyses, since few studies to date use techniques which can quantify parasites of a particular genotype within a mixed infection^20^.

Measuring the frequency of resistance mutations in parasites as well as the prevalence in humans is important because frequencies are more comparable when levels of malaria transmission and the number of clones in infected individuals vary across different areas or time points. Both prevalence and frequency of resistance may also be clinically relevant for IPTp and IPTi. Prevalence is an important indicator of the likelihood of individuals remaining parasite positive after treatment, which appears to be particularly important in pregnant women who are often already infected prior to pregnancy, and where increased duration of placental infection worsens outcomes^21^. In areas with the 581G mutation, one study suggested that SP-IPTp could actually increase parasite densities by clearing wild type parasites and thereby facilitating greater multiplication of these highly resistant parasites^14^, though this was not found elsewhere^15, 22^. However, in areas without the 581G mutation, the ability of SP to reduce parasitaemia and MOI in pregnant women without fully clearing all parasites may reduce anaemia and contribute to preventing low birth weight^6, 23^. If clearing some clones from a multiclonal infection is beneficial, the proportion of parasite clones which are resistant, i.e. the frequency, may be important. The impact of IPT in infants is thought to be mainly due to providing prophylaxis against new infections inoculated by bites from infectious mosquitoes^24, 25^, and this probably also contributes to IPT impact in pregnant women, as evidenced by increased effect of more SP-IPTp doses after initial clearance of infection^7^. Mosquitoes harbour fewer parasite clones than humans, often only a single clone^17, 18, 26^. Therefore, the frequency of resistance in parasites might be a better indicator of the ability of SP to prevent new infections from mosquitoes rather than prevalence of resistance in humans although this has not been evaluated. Many studies have also found that clinical episodes of malaria result from acquiring a new parasite clone, even in individuals already asymptomatically infected with other clones^27–30^. This suggests that clearing particular clones rather than all parasites in an infection has a clinical impact and therefore the frequency of resistant clones as well as the presence of any resistant clone within an infection may matter.

The frequency of resistance in parasites in a survey is often estimated by simply excluding mixed wild type-resistant infections from analysis, and presenting the proportion of remaining individuals who have a pure resistant infection. However, this can mean a considerable proportion of samples are excluded in areas of moderate-high transmission and medium resistance prevalence^19, 31^, and can be biased because the MOI distributions in pure resistant and pure wild type infections are not necessarily the same^19^. Several previous studies have developed other methods for estimating the frequency of resistance. When detailed data are available on the MOI together with presence of resistant and wild type parasites in individual infections, the frequency can be estimated by maximum likelihood^32–35^. These methods are ideal where such detailed data are available, however this is often not the case with routine surveillance data of molecular markers. This issue was noted as an unsolved challenge during previous molecular marker mapping exercises^36, 37^. Others proposed that where individual MOI data were not available, frequencies could be estimated from the observed proportion of mixed resistant-wildtype, all-resistant and all-wild type infections, and the mean population MOI, assuming a plausible MOI distribution (for example, Poisson)^31, 38^. This worked well on simulated data, and was also applied to field data in Uganda^31^. However, even mean MOI data are usually not available from sites with resistance surveillance data, and sometimes only the prevalence of resistance is reported, not the proportion of mixed resistant-wild type infections.

Here, we further develop these previously published methods in order to estimate the frequency of resistance mutations in the parasite population from the reported prevalence of resistance in infected humans in routine surveillance data, first validating our methods using a large, detailed dataset from southern Tanzania^39, 40^. We then update a systematic review of the prevalence of the 540E and 581G mutations in infected humans across Africa and apply our method to also estimate the frequency of these mutations in the parasite population in each area. We map mutation prevalence and frequency and quantify spatiotemporal trends in SP resistance.

## Results

### Distribution of 540E and 581G mutations: systematic review

**Figure 2:**
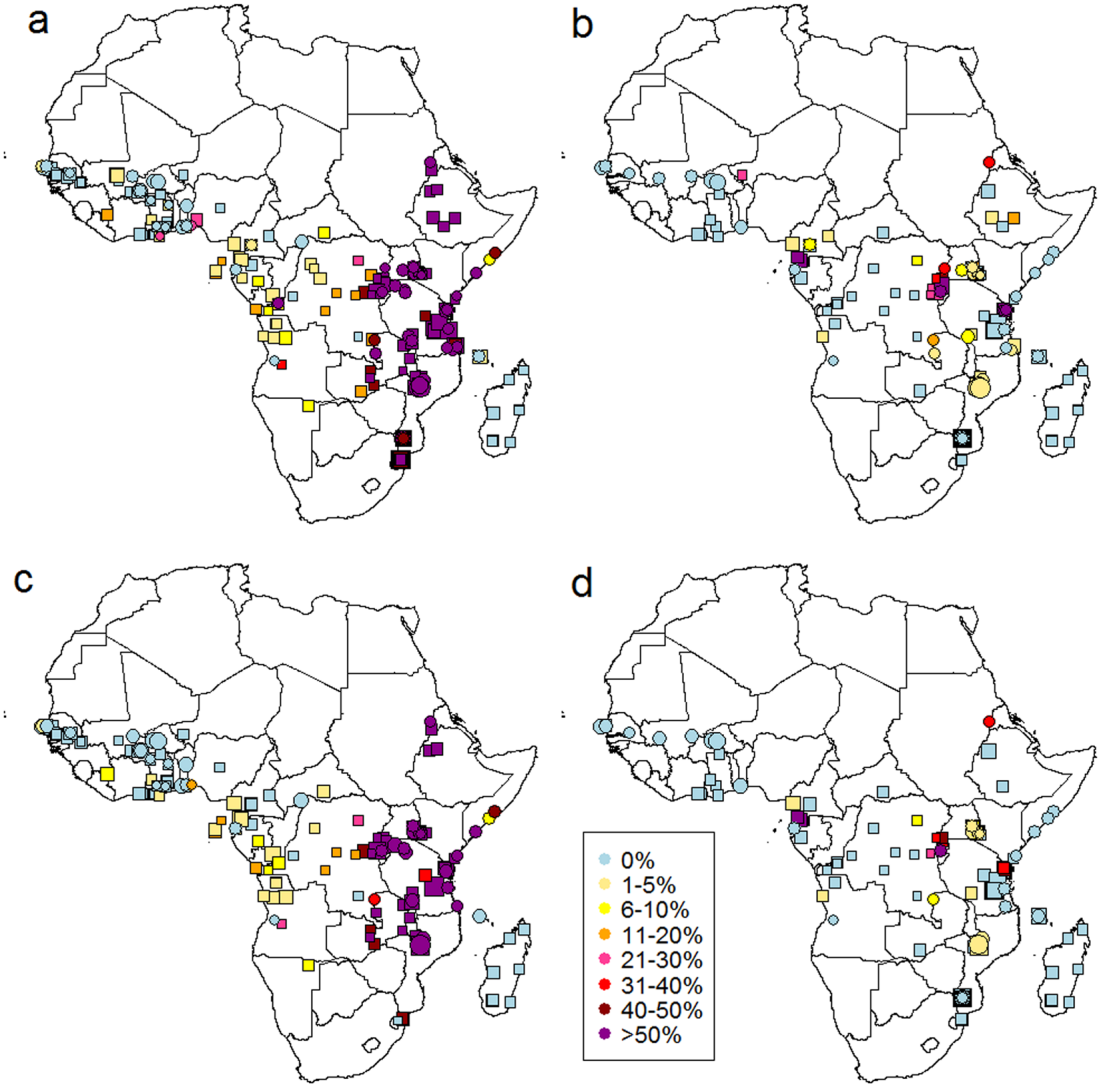
Prevalence of *dhps* (**A**) 540E and (**B**) 581G in infected individuals and estimated frequencies of (**C**) 540E and (**D**) 581G mutations in parasite populations in Africa 2004–2008 (squares) and 2009–2013 (circles) (see also Supplementary data 1 and 2). Data point size is proportional to sample size, with most recent data on top. (**A**) 540E prevalence was assessed in 294 surveys in 32 countries since 2004, of which only 6 did not detect the mutant at all: Burkina Faso, Gambia, Côte d’Ivoire, Madagascar, Niger, Swaziland. Nine countries detected 540E prevalence >50%: Sudan, Somalia, the Republic of the Congo, Tanzania, Malawi, Kenya, DRC, Mozambique, Ethiopia, Rwanda, Uganda and Zambia. (**B**) 581G prevalence was assessed in 147 surveys in 26 countries, of which 13 countries detected the mutation: Angola, Cameroon, Democratic Republic of the Congo, Ethiopia, Equatorial Guinea, Kenya, Malawi, Niger, Rwanda, Sudan, Tanzania, Uganda and Zambia. (**C** and **D**) Frequencies are shown only for surveys which reported the prevalence of mixed infections (27% of total surveys), or estimated frequency by excluding mixed infections from analysis (21%), or had zero prevalence (32%). Surveys which counted mixed infections as mutants (11%), or did not specify how mixed infections were dealt with (8%) were excluded. Frequencies are estimated using Method 5C (Methods section, equation 8), assuming the probability of detecting each clone was 0.54. Maps were generated in R software version 3.1.3^62, 61^ and are based on 180 publications (62 since 2011). Similar patterns are confirmed by an independent review of 35 publications carried out after our review was complete^63^.

We updated a systematic review^41, 42^ of the distribution of *dhps* 540E and 581G mutations in Africa, including all published studies which assessed the prevalence of the mutation in *P. falciparum-*infected individuals in endemic areas (full data available in Supplementary data 1 and 2 and at www.drugresistancemaps.org). We excluded samples of patients recently treated with SP as part of the study. The previous review was completed in 2011 and the most recent samples in that review were collected in 2008 (the average time lag between sample collection and publication is 5 years^41^). The updated review includes studies published up to March 2016, although the most recent surveys reported in those publications were carried out in 2013. The review generated 182 new measures of the 540E mutation and 157 of the 581G mutation (Fig. 2A). Since 2008, three new countries measured a prevalence of 540E >50%: Sudan, Somalia and the Republic of the Congo, in addition to the nine countries already measuring this level of the resistance marker before 2008 (Fig. 2A). In only 6 of 32 countries were all samples wild type at the 540 locus; most were in West Africa where prevalence remained low in the large majority of samples (Fig. 2A). Thirteen of 26 countries detected the 581G mutation (Fig. 2B). All these had already identified it in surveys from 2008 and earlier. Surveillance coverage generally improved, for example in the DRC, but remained sparse in parts of West Africa.

### Estimating resistance frequency from prevalence measures: validation of methods

In the systematic review surveys, the prevalence of mixed resistant-wild type infections among infected individuals varied from 0 to 82%, indicating that the frequency of resistance in the parasite population might be different from the prevalence of resistance in humans in some areas. We sought to estimate frequencies in the parasite populations in each survey from the systematic review data by applying and developing existing methods^19, 31, 32, 35^. The main challenge was that the systematic review data mainly provide only summary measures on the prevalence of *dhps* mutations in humans in each survey, and no information on how many parasite clones were observed per person, whereas most of the existing methods for estimating frequency require detailed data for each individual on mutations and their MOI. Furthermore, the imperfect sensitivity of PCR methods to detect all clones in an infection needed to be taken into account^19^.

**Figure 3:**
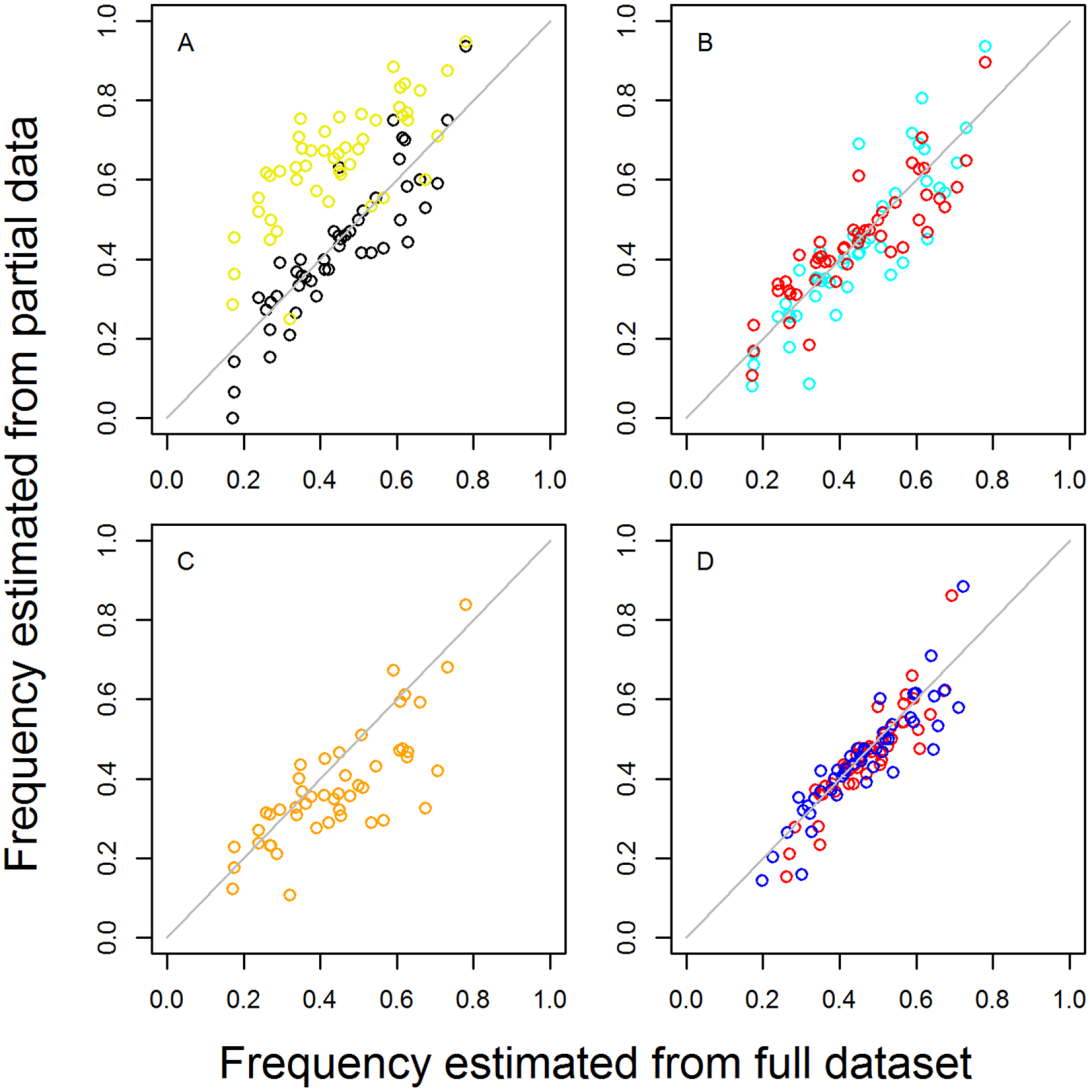
Estimating mutant frequencies in parasite populations: validation of different methods using 540E and MOI data from cross-sectional surveys of 5131 people in 2004 and 2007 in 24 divisions in Tanzania^39^. Ideally, frequencies are estimated using detailed data from all infected individuals on their MOI and whether an infection is pure resistant, pure wild type or mixed. We plot 540E frequencies estimated using only partial data, such as is more often available from routine surveillance of mutations (Methods 3–5), against frequencies estimated from this full dataset for each location (Method 2; x axis on all panels) : (**A**) **black points**: 540E frequencies estimated by excluding mixed infections (Method 1A vs Method 2A); **yellow points:** for comparison we also show the prevalence of any 540E mutation among infected individuals (**B**) **blue points**: 540E frequencies estimated from data on mixed infections and mean population MOI (Method 3A vs Method 2A); **red points: 540E frequencies estimated from data on mixed infections but** with no MOI data (Method 5A vs Method 2A), instead estimating mean MOI using the Malaria Atlas Project slide prevalence and the relationship in Fig. 4 (**C**) **orange points:** 540E frequencies estimated with no MOI data nor data on mixed infections, using data on resistance prevalence only (Method 8A vs Method 2A) (**D**) **red points:** 540E frequencies estimated with data on mixed infections but no MOI data when detection of clones is imperfect, either assuming clones are missed in high MOI infections (Method 5B vs Method 2B) or (blue points) clones have a constant probability of being missed in any infection (Method 5C vs Method 2C).

First, we compared and validated methods for estimating frequencies from prevalence data using a test dataset for which we have detailed data for each individual on resistance markers and MOI. The test dataset is from 5191 samples from cross-sectional surveys of individuals of all ages in 24 district level clusters in Tanzania in 2004 and 2007^39^ Individuals were not selected based on symptoms of malaria. We chose the 540E mutation for analysis because its frequency varied considerably across the study area and by year. We compared several methods and assumptions used for estimating frequency, each of which are defined fully in the Methods section and assigned a number. At first, for simplicity, we assumed that 100% of parasite clones are detected (this assumption is denoted by the letter A after the method number). Firstly, we estimated the frequency of 540E in each cluster and year using an existing method by Hastings *et al*. (Method 2A)^32^ which utilises the full information on resistance markers and MOI for each individual in the Tanzanian test dataset. We found that the estimated frequencies in the Tanzanian parasite populations were consistently lower than the prevalence of resistance mutations in humans, as expected (Figs 1 and 3A).

We next used several adjusted methods to estimate frequency without using the full data, to resemble systematic review data, to see how accurate these estimates can be, for example when we lack information on MOI or mixed infections. Frequency is commonly estimated simply by excluding mixed wild type-resistant infections from analysis and calculating the proportion of the remaining infections that are resistant (Method 1A). In the Tanzania test dataset this method gave relatively similar frequencies to those estimated from analysis of the full individual information (Method 2A, Fig. 3A and Table 1) but has the disadvantage that a considerable number of mixed samples may be excluded from the analysis, up to ~60% per survey in this test dataset (Figure S2). There was also some bias in this frequency estimate because the mean MOI of pure resistant versus pure wildtype infections was 2.7 versus 3.3, respectively, which the method does not take into account. We next tried estimating frequencies in the Tanzanian dataset using the proportion of pure resistant, pure wild type and mixed wild type-resistant infections, but without using the individual MOI data for each person. Instead we used the method of Taylor *et al*.^31^, where it is assumed that we know only the population mean MOI, and we allow for the variation in MOI across infected people by assuming a Poisson distribution of the number of clones per person (Method 3A). Frequencies estimated using this method 3A correlated well with the estimates made using the full individual MOI data (Method 2A, Fig. 3B and Table 1).

**Table 1:** Estimating mutant frequencies using different methods: validation using a test dataset from Tanzania^39^. Frequencies of the 540E mutation are estimated in 24 areas in 2 different years.

Method	Data required				Assumptions		Mean squared error	Reference for Method
	Mixed infections	Mean population MOI	Slide prevalence	Location	Assumption about detection of parasite clones	Other		
5B	√			√	Imperfect MOI-dependent		0.0031	This paper extended from^32^
5C	√			√	Imperfect MOI-independent		0.0037	This paper
7A	√			√	100%	mean MOI varied during fitting	0.0061	This paper extended from^31^
5A	√			√	100%		0.0062	This paper extended from^31^
6A	√			√	100%	Assume negative binomial MOI distribution	0.0071	This paper extended from^31^
3A	√	√			100%		0.0071	31
4A	√		√		100%		0.0083	This paper extended from^31^
8A				√	100%		0.0140	This paper extended from^31^

**Figure 4:**
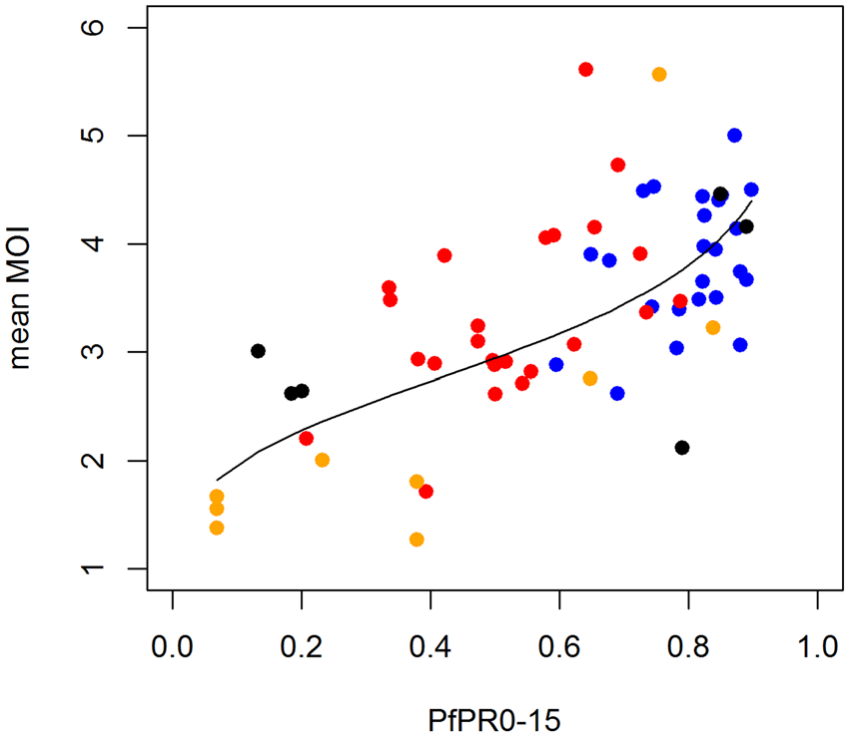
Relationship between slide-prevalence of infection in 0–15 year olds (*Pf*PR0–15) and the mean multiplicity of infection (MOI) in infected individuals in 63 sites. Data (points) and model fit (black line). Data are: (blue) asymptomatic individuals in the test datset in Tanzania in 2004 and (red) 2007;^58^ (orange) data from a previous review in multiple sites^43^ containing both symptomatic and asymptomatic individuals; and (black) from symptomatic patients in Uganda^64^. 48 of the measures had simultaneous measures of slide-prevalence, while the other 15 were paired with an alternative measure of transmission intensity, the entomological inoculation rate (EIR, number of infectious bites per person per year). We converted the EIR values to slide-prevalence values in 0–15 year olds, using a previously published relationship^60^. Model fit (black line) is from linear regression of log MOI against the log odds of *Pf*PR0–15. The best fitting relationship is log MOI = 1.082 + 0.185*logit(*Pf*PR0–15). This is further confirmed with a similar relationship fitted in a separate review and mathematical modelling paper published after our analysis was complete^65^.

We compare frequencies estimated from the full dataset with information on MOI and resistance markers for each individual (Method 2, see Methods) with frequencies estimated from partial summary data using different methods. We show mean squared error to quantify the difference between the two sets of estimates in each comparison. The methods are ordered by increasing mean squared error; lowest indicates more similar estimates. The column ‘mixed infections’ indicates that data on the proportion of mixed wild type-resistance infections was used to estimate frequencies. ‘Slide prevalence’ indicates that mean MOI was estimated using the relationship in Fig. 4. ‘Location’ indicates that longitude and latitude of the survey location were used to obtain estimates of slide-prevalence from the Malaria Atlas Project, in order to estimate mean MOI using Fig. 4 ^45^. Where imperfect detection of parasite clones was assumed, this was included when fitting to both the full and partial datasets (letters A to C after the Method number denote assumption about detection; see Methods. For example, in the first row, method 5B is compared to method 2B, in the 2^nd^ row method 5C is compared to method 2C etc).

However, even mean population MOI estimates are usually not available in the surveys in our systematic review. Therefore, we also tried estimating frequencies without any MOI data (Methods 4A and 5A). Instead we used the fact that the average number of parasite clones per person increases with malaria transmission intensity^43^, which is more commonly measured. To characterise this relationship, we assembled 63 paired measures of mean MOI in infected individuals and malaria transmission intensity (Fig. 4). We used slide-prevalence of malaria in 2–10 year olds as the measure of transmission intensity, since this is most widely available^44^. A linear relationship of log mean MOI with logit slide prevalence in 2–10 year olds (p < 0.001) gave a good fit to the data (Fig. 4). Some molecular marker surveys measure slide-prevalence, and therefore this can be directly used to estimate mean MOI, but if they do not, slide-prevalence estimates are available from the Malaria Atlas Project across Africa for each year from 2000–2015 at a 5 × 5 km resolution^44, 45^. We estimated mean MOI for each cluster and year in the Tanzania survey test dataset, using either slide-prevalence measured during the survey (Method 4A) or estimated slide prevalence from the Malaria-Atlas Project^44^ in the same year as the survey (Method 5A). These mean MOI values were then used to generate a distribution of MOI in the population, assuming a Poisson distribution as before. Frequencies estimated using both these methods (Methods 4A & 5A) correlated very well with the estimates made using the full test dataset (Fig. 3B and Table 1). Using estimated rather than true mean MOI (Fig. 3B, Method 3A)) or the true slide prevalence (Method 4A) made negligible difference (Table 1), despite neither the MOI or slide-prevalence estimates being very well correlated with their true values (Supplementary Fig. S1). Assuming a negative binomial distribution for MOI instead of a Poisson distribution (Method 6), or allowing the mean MOI to vary during fitting (Method 7) or also produced negligible difference in frequency estimates (Table 1). Together, the comparison of methods 2–7 indicate that the estimated frequencies are not highly sensitive to the assumed MOI distribution, confirming previous results^31, 35^. This shows that good frequency estimates can be obtained without needing MOI data for each survey.

We next tried estimating frequencies using data from all individuals, but assumed that we only know the prevalence of infections carrying at least one resistance marker (Method 8A), not the prevalence of mixed wild type-resistant infections, as is often the case with published molecular marker surveys (mixed infections may be counted as resistant). However, frequencies estimated using only this information differed considerably from frequencies estimated from the full individual dataset (Method 2A) (Fig. 3C and Table 1), indicating that knowing the proportion of mixed infections is important.

The analyses presented so far assume 100% detection of parasite clones. We further considered that some parasite clones may not be detected during laboratory measurement, for example due to low densities. This was found to be important in a previous analysis to improve fit to the data^19^. We incorporated imperfect detectability into the frequency estimation method which uses the full individual data (Method 2), as a baseline for comparison. We also chose to incorporate detectability into method 5 which estimated frequencies well using only information on the proportion of mixed infections and the Malaria-Atlas-Project-estimated slide prevalence, i.e. with the most parsimonious assumptions and data. We tested 3 different assumptions about detectability: (A) that 100% of clones are detected, as described above, (B) following a published method by Hastings *et al*.^19^, that resistant or wild type clones in an infection are not detected if they constitute less than a given proportion (the ‘genotyping sensitivity limit’) of the total clones or (C) that the probability of missing any clone is constant regardless of the MOI of an infection (similarly to Ken-Dror *et al*.^35^). We use these letters A, B and C to denote these different detectability assumptions. We first compared these different detectability assumptions when the frequencies were estimated from the full individual Tanzanian test dataset. Assumption C gave the best fit of the model to the test dataset (Method 2C), with the previously published method^19^ (Method 2B) fitting next best and the assumption of 100% detection (Method 2A) fitting worst (log likelihoods: −1616, −1772 and −2110, respectively). The probability of detecting each clone estimated by method 2C was 0.54 (95% CI 0.25–0.77), and the best fitting genotyping sensitivity limit in the published method (2B) was 0.33. Including imperfect detection improves the fit of the model to the proportion of mixed infections (Supplementary Fig. S2; Methods 2A, 2B and 2C). Estimating frequency using imperfect detection models again worked well even if no MOI or slide-prevalence data were used (Methods 5B and 5C) (Fig. 3D and Table 1). In addition, we tried three further plausible extensions to the model with imperfect detection: (1) assuming that the probability of missing a clone increases linearly with MOI; (2) assuming that in multiclonal infections, a single dominant clone is detected with a higher probability than the other clones; (3) assuming there is geographical heterogeneity in the proportion of resistant clones. All these extensions slightly improved the fit of the model to the data (Supplementary methods), but as none clearly outperformed the others on this test dataset, we did not further apply these methods in this paper although they could be applied to detailed individual data in other settings.

### 540E and 581G frequency maps

Having tested and validated methods for estimating resistance frequencies in parasite populations, we applied these to the systematic review survey data on the prevalence of resistance markers in infected humans to generate new frequency maps of 540E and 581G (Fig. 2C and D). Our analysis validation on the Tanzanian test dataset showed that frequencies can be estimated well without MOI data, instead estimating mean MOI for each survey population using a relationship between mean MOI and Malaria Atlas project-estimated slide-prevalence and assuming a Poisson distribution for MOI. However, it was important to have data on the proportion of mixed wild type-resistant infections, without which frequencies were poorly estimated. The frequency estimation model which fit the test data best assumed that detection of parasite clones is imperfect and does not depend on MOI. We therefore applied the analysis method which incorporated all these aspects (Method 5C; see Methods section) to estimate frequencies in all surveys which had mutation prevalence greater than zero and which reported the prevalence of mixed infections (n = 126 surveys for 540E, n = 38 for 581G). R code for estimating frequencies is provided in the Supplementary Information. Where the prevalence of resistance was zero, we assumed the frequency was also zero. The maps (Fig. 2C and D) also include sites where the original study estimated frequency by simply excluding mixed infections from analysis. For sensitivity analysis, we also varied the probability of detecting parasite clones, since the sensitivity of PCR methods is likely to vary between studies.

**Figure 5:**
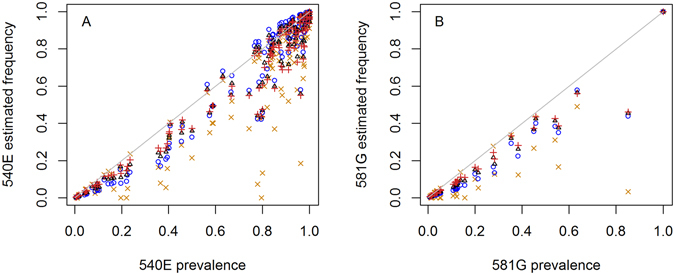
Prevalence of (**A**) 540E and (**B**) 581G mutations in the systematic review data compared with frequencies in the parasite population estimated by different methods: simple counting of mutant samples excluding mixed infections (orange crosses; Method 1A); by assuming 100% detection of clones (blue circles; Method 5A); by assuming detection is independent of MOI (Method 5C) with a probability of detection per clone of 0.54 (black triangles); or 0.3 (red vertical crosses). Surveys were included in these figures if they detected the mutant and reported the prevalence of mixed wild type-resistant infections.

Frequencies of 540E and 581G in the parasite population gave a very different picture from the prevalence of 540E and 581G in humans in some surveys, with up to 40% absolute difference between measures, whilst in other surveys, the difference was small or zero (Fig. 5). When prevalence is low (<~5%), there are only small differences from frequency estimates. When prevalence is high, for example >90%, in some surveys this is because the mutation is truly approaching fixation in the parasite population, and then frequency estimates are close to prevalence. In other surveys, this high observed prevalence simply reflects a high level of mixed infections in the human population and frequency is at an intermediate level (Fig. 5). At intermediate 540E prevalence (>10% and <90%, n = 56 surveys) the frequency was on average 9–10% lower than prevalence (depending on detection assumption). The maximum observed difference was 37%. Similarly, at intermediate prevalence of 581G (n = 20), frequency was 8–11% lower than prevalence. Varying assumptions about the sensitivity of detection of parasite clones did not change the estimated frequencies substantially (mean difference of 2.4% when changing sensitivity from 100% to 30% in Method 5C), although assuming imperfect detection improved the fit of the model to the proportion of mixed infections in the surveys (Supplementary Fig. S3).

If specified thresholds were used to classify areas as suitable for particular interventions, using frequencies rather than prevalence could give very different results. For example, of 38 surveys, 22 surveys had 581G prevalence over 10% whilst only 14–16 had 581G frequency over 10%. It is not known whether resistance frequencies correlate with outcomes of SP-based interventions such as IPT, but for a hypothetical example, had a 10% 581G frequency threshold been used to decide a local policy at the time they were done, an extra one third of survey results would have been under the threshold compared with a 10% 581G prevalence threshold. These particular results were from areas with high malaria transmission, with an estimated mean slide prevalence in 2–10 year olds (*Pf*PR2–10) of 46% (range 28–64%) at the time of the surveys. Interestingly, using frequency rather than prevalence for a 50% 540E threshold (the current IPTi policy recommendation) would only cause 6 out of 126 survey results to be additionally under the threshold. This is because resistance generally spreads rapidly at intermediate prevalence so is only at these levels for a short time with only 13% of surveys showing a prevalence between 30–70% (Fig. 5A).

### Spatiotemporal trends in dhps mutations

**Figure 6:**
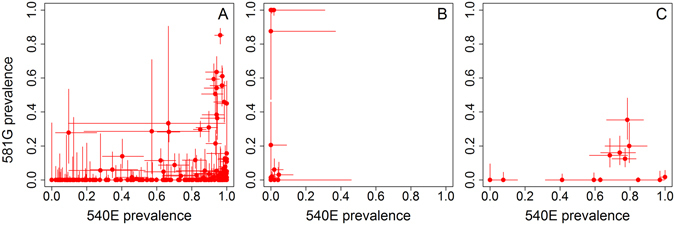
Relationship between prevalence of 540E and 581G mutations when measured in the same individuals in (**A**) Eastern and Southern Africa (n = 172), (**B**) West Africa (n = 50), (**C**) Northeast Africa (n = 15) using surveys from 1988–2013. Mixed infections contribute to some of these measures, therefore the haplotype is unknown for most samples (i.e. the two mutations could be present together on one parasite or separately on different parasites). The sample size for assessing the prevalence of the two mutations was not always identical, usually due to different PCR success rates. Data in Eastern and Southern Africa were from the Democratic Republic of the Congo, Kenya, Mozambique, Malawi, Rwanda, Tanzania, Uganda, South Africa and Zambia; in West Africa from Angola, Benin, Burkina Faso, Côte d’Ivoire, Cameroon, Ghana, Equatorial Guinea, Mali, Mauritania, Niger and Senegal; and Northeast Africa from Ethiopia, Sudan and Somalia.

Marked differences were seen in the relationship between 540E and 581G in different regions (Fig. 6). In Eastern and Southern Africa, the 581G mutant is much more common where 540E prevalence is high: 52/58 of surveys which detected the 581G mutation had >60% prevalence of the 540E mutation (Fig. 6A). North-east Africa showed a similar relationship (Fig. 6C) despite the likely different genetic origin of these mutations^46, 47^. However, in West Africa, 581G was detected in 20% of surveys despite 540E prevalence and frequency being under 5% in all surveys in this region. 581G was also detected in areas where the 540E mutation was absent (Fig. 6B).

**Figure 7:**
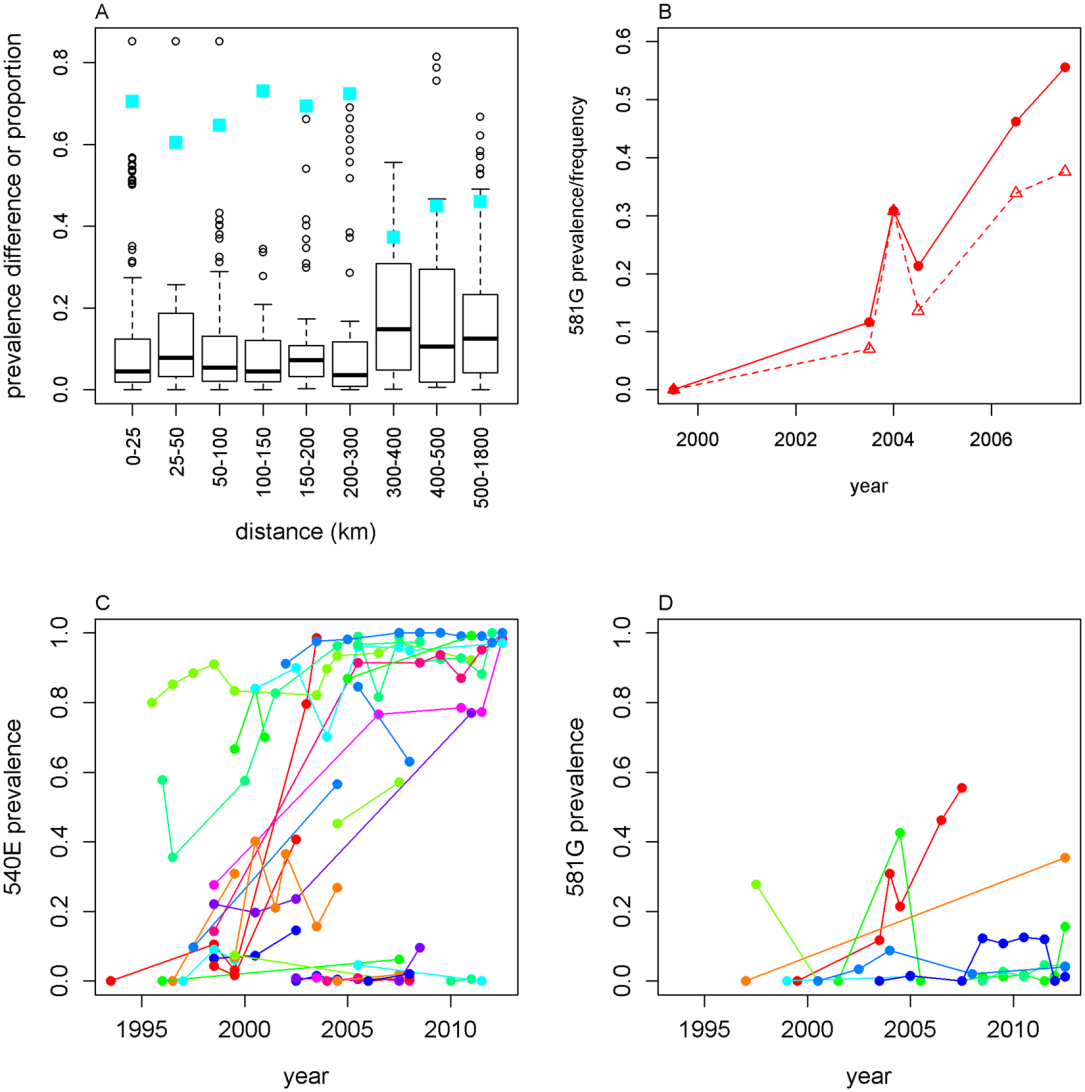
Spatiotemporal variation in 540E and 581G prevalence. (**A**) Different spatial scales. We compared prevalence of the same mutation in the same country between surveys done within 1 year of each other. We plot the pairwise distance between the surveys against the absolute difference in prevalence of the mutation. Blue squares indicate the proportion of survey pairs in which the difference in mutation prevalence was less than 10%. (**B**) Comparison of 581G frequency (triangles, dashed line) and prevalence (circles, solid line) in Tanga, Tanzania. Temporal trends in (**C**) 540E and (**D**) 581G prevalence. We compared successive measures within the same first administrative area which were <100km apart. Supplementary Fig. S4 shows the same results for frequency measures. Comparisons of two zero prevalence measures are excluded in all panels.

The prevalence of mutations within countries was relatively similar for measures taken up to 300 km apart within a year of each other, however at greater distances the difference increased (Fig. 7). Frequencies were overall marginally more consistent over space than prevalence measures (mean squared difference of pairwise comparisons within countries being 0.044 for frequencies, versus 0.047 for prevalence measures) (Supplementary Fig. S4A). To look at changes in mutation prevalence and frequency over time, we grouped data by the largest subnational regions of each country (first administrative areas). In areas which had measures both before 2009 and afterwards (n = 35 for 540E and 17 for 581 G), 540E prevalence increased in 60% of areas, decreased in 20%, and stayed at zero in 20%. 581 G prevalence decreased in only 6% of areas, increased in 59%, and stayed at zero in 35%. The rate of change of mutation frequency or prevalence was highly variable between locations (Fig. 7C and D). Pairwise comparison of successive time points within each area showed a median increase in 540E prevalence of 5.2% (interquartile range (IQR) 0.2, 16.0%) per year (excluding pairs where the prevalence was zero at both times or already >90%). In some areas, frequency measures revealed different temporal patterns of resistance. For example, in Tanga, Tanzania, the frequency of the 581 G mutation increased more slowly than the prevalence of the mutation (Fig. 7B). In this area, the prevalence of the 540E mutation appeared to be close to saturation as early as 1995, remaining between 80–90% for 10 years. However the estimated 540 frequencies suggest only 55% of the parasite population had the mutation in 1995 and it was still increasing in frequency for the next 10 years with some fluctuations (Fig. 7C and Supplementary Fig. S4, light green lines). The median increase in 540E prevalence was generally slower when the mutation was at low levels: 1.8% per year when frequency began at <10%, versus 6.1% increase per year when frequency began at levels >10% but <90%, consistent with the expected ‘S’ shaped growth curve of resistant parasites (e.g. as shown in Smith *et al*.^48^). The median rate of change of 581 G prevalence was lower than 540E at 1.1% (IQR −0.8, 3.0%), but this may reflect low initial prevalence (Fig. 7D). There were no clear differences in temporal trends by region or by calendar time.

## Discussion

We find evidence for continued spread of *dhps* 540E and 581 G mutations in Africa since 2008, despite SP being withdrawn as first line treatment in the majority of countries before this time. Three new countries detected 540E at >50%. 581 G was not detected in any new countries since 2008, but the prevalence of both mutations increased in the majority of countries which had already detected these markers prior to 2008. These findings indicate ongoing SP drug pressure, which may in part arise from IPTp and seasonal malaria chemoprophylaxis programmes, although IPTi has not had wide uptake^49^ and IPTp coverage remains poor in many areas^50^. However, recent market surveys have shown continued high use of SP in several African countries, particularly in the private sector, with for example SP constituting 42% of all antimalarials purchased in Tanzania in 2014^51–53^, suggesting it is still widely used for uncomplicated malaria treatment. The continued spread highlights the need for ongoing surveillance, since IPTp with SP is being scaled up across Africa^9, 49, 50^. Our results suggest that sentinel sites could be spaced a maximum of 300km apart, and that surveillance should be conducted more frequently when mutation prevalence exceeds ~10% (but is not yet fixed).

We show that frequencies of resistance in the parasite population could be estimated well from standard aggregate survey data on resistance markers in infected humans (see Supplementary Information for the full R code), without individual data or MOI data, as long as the proportion of mixed wild type-resistant infections was reported. We mapped *dhps* mutation frequencies, giving a clearer picture of what is happening in the parasite population and enabling comparison across areas and time points with different transmission intensity and MOI distributions. Current IPTi policy is based on the prevalence of resistance markers in infected individuals, and the same is suggested for IPTp policy^16^. It will be critical to further characterise the relationships between clinical impact of IPTi and IPTp with SP and *dhfr* and *dhps* mutations and the biological mechanism of SP action^54^, and ongoing studies are addressing this issue. In the meantime, high prevalence of resistance markers may preclude implementation of IPTi (and potentially IPTp) in some areas. The clinical relevance of mutation frequencies as opposed to prevalence in humans requires further research (see Introduction for more discussion of this issue). We suggest that frequency might be an important indicator of drug prophylactic effect given the prevalence of monoclonal infections within mosquitoes, as well as indicating the chance of clearing particular clones within mixed infections, but this needs to be better characterised. Given these uncertainties, we suggest it would be worth re-evaluating data to inform whether frequency thresholds as well as prevalence thresholds could be useful for policy guidelines. Mixed wild type-resistant infections, which create the difference between prevalence and frequency measures, are most common in high transmission settings, exactly where IPTp and IPTi have the highest public health impact. Frequency provides a lower threshold, as it is equal to or lower than prevalence, therefore the intervention would be recommended in more areas. For example, in our systematic review, we found that 33% more surveys had 581 G mutations under a 10% frequency threshold compared with a 10% 581 G prevalence threshold. However more research would be needed to identify whether frequencies are informative and if so, what a suitable frequency threshold would be.

Whilst using frequency measures increases the comparability of surveys where transmission intensities were different, there are additional factors that we did not control for. The age of individuals in each survey varied considerably and is likely to influence the probability that someone carries resistant parasites, since intake of treatment is different across age groups^39^. Furthermore MOI varies by age^55^. Another potential confounder is that some surveys include largely asymptomatic malaria infections in the community, whilst others recruit patients with symptomatic malaria. Clinical samples may have higher levels of resistance, probably reflecting treatment in the informal sector before seeking care. Further work to quantify the impact of these other factors would be beneficial to standardise resistance measures. The test dataset from Tanzania that we used to validate our methods for estimating frequencies was a survey of asymptomatically infected individuals of all ages. We cannot be sure how accurate the method is when applied to different samples. However, similar methods worked well on clinical samples in other settings^31^.

We did not estimate haplotypes at the 540 and 581 loci because we did not have individual data and the degree of linkage disequilibrium is unknown. In East Africa, 581 G usually occurs together with 540E^47^, but in West Africa, 581 G was detected in the absence of 540E. The public health implications of the latter genotype are unclear and further research is required to characterise its phenotype. We also did not map *dhfr* genes, since these are not the focus of current policy^10^ and few studies reported the full *dhfr* and *dhps* haplotypes. Several methods have been developed to estimate haplotypes of parasites, which employ efficient methods such as Markov-chain Monte Carlo or Expectation-Maximization algorithms to maximise the likelihood^31, 33, 35^, but these all require individual patient data on MOI and multiple resistance markers. Ideally future resistance surveillance studies would measure MOI and estimate frequencies as part of the routine analysis.

Rapid advances in genetics and genetic analysis are improving techniques for measuring MOI^56^ and resistance frequencies within infected individuals, enabling quantification of parasites with different genotypes and distinguishing haplotypes in mixed infections^57, 58^. Whole genome data can be used to estimate MOI, for example using the estMOI software^59^. These techniques would provide extremely useful data but may not be available for routine surveillance of resistance markers for some time. The analyses presented in this paper show that robust estimation of frequencies of single SNPs is possible from routine surveillance data without more resource-intensive molecular methods. These methods can be applied in future to standardise data on other resistance markers, in particular artemisinin resistance-associated mutations at *kelch 13* and mutations conferring resistance to partner drugs in artemisinin combinations therapies, such as the emerging piperaquine-resistant strains in Cambodia which represent a severe public health threat.

## Methods

### Systematic review

We searched PubMed in March 2016 using the terms “malaria” and at least one of “dhps”, “pfdhps”, “540” or “581”, screening all studies published from 2011 onwards, when the previous systematic review was completed. We included studies which reported the prevalence or frequency of the 540E and/or 581G mutation in *P. falciparum*-infected individuals in a specific geographical area and the date of sampling. We collected information on the location, date, sample size, reporting of mixed infections and age range.

### Data

Full details of the test dataset used to compare different methods for estimating frequency are given in Pearce *et al*.^39^. In brief, survey data were from cross-sectional surveys conducted in 2004 and 2007 during a cluster-randomized trial of IPT with SP in infants. The intervention was implemented in 2005. Blood samples were collected on filter paper, and mutations at codon 540 of the *dhps* gene were detected by polymerase chain reaction (PCR) sequence-specific oligonucleotide probing (SSOP). Infections were counted as mixed if peaks could be detected above the noise threshold. MOI was assessed by polymorphisms in microsatellite markers PolyA, PfPk2, and TA109, and was calculated as the maximum number of observed variants.

Estimates of slide-prevalence in 2–10 year olds were taken from raster files published by the Malaria Atlas Project^45^. For each of the 24 clusters in the IPTi trial and for each survey in the systematic review, estimates were extracted using the year and longitude-latitude of the survey location. In order to estimate mean MOI in the area using the relationship in Fig. 4, these were converted to estimates of slide-prevalence in 0–15 year olds using a previously published model^60^.

### Analysis: estimating frequency

The frequency of resistance *p* is defined as the proportion of parasite clones which have a resistance marker. Following the equations by Hastings *et al*.^19^, this is calculated as:

1 $$p=\sum _{i}\frac{{c}_{i}}{{n}_{i}}$$

where *c*_*i*_ is the number of resistant clones and *n*_*i*_ is the total number of clones in individual *i*. The prevalence of resistance *Prew*(*res*) is defined as the proportion of infected humans who are carrying at least one resistant parasite clone. The relationship between the prevalence of resistance in humans *Prev*(*res*) and the frequency of resistance in the parasite population p, following Hastings *et al*.^19^ is:

2 $$Prev(res)=\sum _{n=1}^{{n}_{max}}{d}_{n}[1-{(1-p)}^{n}]$$

where *n* is the MOI of each individual infection, *p* is the frequency of resistance and *d*_*n*_ is the proportion of samples which have an MOI of n. The prevalence of the wildtype *Prev*(*wt*) is calculated using the same equation, replacing the frequency of resistance *p* with the frequency of the wild type (1 − *p*). The proportion of human with mixed wildtype-resistant infections *P*(*mix*) is calculated from these as:

3 $$P(mix)=Prev(res)+Prev(wt)-1$$

The proportion of infections with all wild type *P*(*wt*) or all resistant parasites *P*(*res*) is calculated by subtracting *P*(*mix*) from *Prev*(*wt*) or *Prev*(*res*), respectively.

The frequency which maximised the likelihood of the observed prevalence of a molecular marker in any given survey was estimated, contrasting methods which make different assumptions and also those which use detailed individual datasets versus those which use various summary measures more often available from published surveys. The details of each method and the data used by each are as follows:

#### Method 1

Frequency is estimated simply by excluding mixed wild type-resistance infections from analysis and calculating the proportion of infections which contain only resistant clones (all-resistant infections). This assumes that the distribution of MOI is the same in all-resistant as in all-wild type infections. It results in a lower sample size for estimating frequency.

#### Method 2

The published Malhaplofreq algorithm^32^ which uses the full data where MOI and presence/absence of resistance markers is known for each individual (this was applied to the test dataset from Tanzania only). Here *d*_*n*_ is simply the observed proportion of individuals with a given MOI in the data.

#### Method 3

This method can be applied when only the prevalence of resistant and mixed infections, and the mean MOI in the study population are known. Here, *d*_*n*_ is assumed to follow a zero-truncated Poisson distribution, which would be observed when individuals are each infected at the same average rate. The zero-truncation allows for only analysing data from infected individuals. We fit the equations for *P*(*res*), *P*(*wt*) and *P*(*mix*) described above to the observed proportion of all-resistant, all-wildtype and mixed infections in each survey, respectively, using multinomial likelihood. These assumptions are similar to those used by Taylor *et al*.^31^ but because we analyse only one SNP at a time, we could use standard methods to maximise the likelihood.

#### Method 4

As method 3, except the sample mean MOI is unknown whilst the local slide-prevalence of malaria is known. We estimate mean MOI using the relationship in Fig. 4.

#### Method 5

As method 3, but the mean MOI and the local slide prevalence are unknown. We use estimated slide-prevalence from the Malaria Atlas Project based on the location and year of the survey^44^.

#### Method 6

As method 5, but *d*_*n*_ is assumed to follow a zero-truncated negative binomial distribution where some individuals are at higher risk of infection than others, assuming a relatively high level of variation (dispersion parameter = 0.5).

#### Method 7

As method 5, but the mean MOI, as well as the frequency, is allowed to vary during the fitting procedure.

#### Method 8

As method 5, but the proportion of mixed infections is unknown, only the prevalence of resistance (counting mixed infections as resistant) is known. We fit equation 2 to the observed prevalence of resistance in each survey using binomial likelihood.

For each of the methods 2–8, we further assessed three different assumptions about the sensitivity of detection of parasite clones:

A) Perfect 100% sensitivity.

B) That the probability of detecting a clone is dependent on the MOI of the infection, as described in the method by Hastings *et al*.^32^. Minority clones present at a density under a ‘genotyping sensitivity limit’ in mixed infections are assumed to remain undetected. This method modifies equation 2 to compute prevalence, calculating which clones would be detected in each possible SNP combination:

4 $$Prev(res)=\sum _{n=1}^{n\_max}{d}_{n}\sum _{c=1}^{n}(\begin{array}{c}n\\ c\end{array}){p}^{c}{(1-p)}^{n-c}v$$

where *v* is 0 if c/n is less than the genotyping sensitivity limit and 1 otherwise. We varied the assumed genotyping limit simultaneously with the frequency to maximise the likelihood of the observed data.

C) That the probability of detecting a clone is independent of the MOI of the infection. We assumed that each clone, resistant or wild type, has a certain probability *q* of being detected, and calculated the proportion of resistant infections as follows. The probability of an all-resistant infection for a given value of *c* is the probability that all susceptible clones are missing multiplied by the probability that at least one resistant clone is non-missing

5 $$P(res|n,c)={(1-q)}^{n-c}(1-{(1-q)}^{c})$$

Summing over all possible values of *c*

6 $$P(res|n)=\sum _{c=1}^{n}(\begin{array}{c}n\\ c\end{array}){p}^{c}{(1-p)}^{n-c}({(1-q)}^{n-c}-{(1-q)}^{n})$$

Equation 6 can be simplified (see Supplementary methods) to:

7 $$P(res|n)={(p+(1-p)(1-q))}^{n}-{(1-q)}^{n}$$

and when the assumption is made of a Poisson-distributed MOI with mean µ:

8 $$P(res)={e}^{\mu (p+(1-p)(1-q)-1)}-{e}^{-\mu q}$$

When fitting to the Tanzanian dataset, we estimated *p* and *q* simultaneously, allowing *p* to be different in each cluster and year but assuming *q* was constant. We obtained confidence intervals using profile likelihood. The sensitivity of methods used to detect MOI is much higher than the methods used to detect resistance markers^39^, and therefore we assumed no effect of imperfect detection on MOI measures.

For each analysis we specify which of the methods 1–8 is used by its number, and which detectability assumption is used by the letters A, B and C.

## Electronic supplementary material

Supplementary Information

Supplementary Dataset 1

Supplementary Dataset 2

Supplementary Dataset 3
