# Supplementary material for: Mapping sulphadoxine-pyrimethamine-resistant Plasmodium falciparum malaria in infected humans and in parasite populations in Africa
Source: Sci Rep. 2017 Aug 7;7:7389. doi: 10.1038/s41598-017-06708-9 (PMC5547055; doi:10.1038/s41598-017-06708-9)
Supplement: Supplementary file 1 — Supplementary Information [file 41598_2017_6708_MOESM1_ESM.doc]

Mapping sulphadoxine-pyrimethamine-resistant *Plasmodium falciparum* malaria in infected humans and in parasite populations in Africa

**LC Okell, JT Griffin, C Roper.**

## Supplementary Information

**Supplementary Methods**

The following gives the full derivation for equations 7 and 8 in the main text. Suppose the MOI *n* follows a distribution with probabilities

$$P(n), n=0,1,2,...$$

For the parameter estimates here and in the main text we assumed a Poisson distribution, but for completeness formulae are also stated for the negative binomial distribution.

Each clone is detected with probability *q*. Conditional on a given value of $n$, with $n>0$, the probabilities that a sample has all resistant, all susceptible, mixed or all missing clones are as follows. Let $c$be the number of resistant clones. For a given value of $c$, the probability of an all-resistant sample is the probability that all susceptible clones are missing multiplied by the probability that at least one resistant clone is non-missing

$$P(res|n,c)=(1-q)^{n-c}\left(1-(1-q)^{c}\right)=(1-q)^{n-c}-(1-q)^{n}$$

Summing over all possible values of $c$

$$\begin{matrix} P(res|n)=\sum_{c=1}^{n} \left(\begin{matrix} n \\ c \end{matrix}\right)p^{c}(1-p)^{n-c}\left((1-q)^{n-c}-(1-q)^{n}\right) \\ =\sum_{c=0}^{n} \left(\begin{matrix} n \\ c \end{matrix}\right)p^{c}(1-p)^{n-c}\left((1-q)^{n-c}-(1-q)^{n}\right) \\ =\left(p+(1-p)(1-q)\right)^{n}-(1-q)^{n} \end{matrix}$$

This uses the fact that the term for $c=0$ is $\left((1-q)^{n}-(1-q)^{n}\right)=0$, and that $\sum_{c=0}^{n} \left(\begin{matrix} n \\ c \end{matrix}\right)a^{c}b^{n-c}=\left(a+b\right)^{n}$ for any $a,b$.

This probability can also be inferred as the probability that each clone is either resistant with probability $p$or is susceptible and missing with probability $(1-p)(1-q)$, minus the probability that all clones are missing.

Similarly, the probability of an all-susceptible sample is found just by swapping $p$and $1-p$with each other

$$P(susc|n)=\left((1-p)+p(1-q)\right)^{n}-(1-q)^{n}$$

The probabilities of all-missing and mixed samples are

$$\begin{matrix} P(miss|n)=(1-q)^{n} \\ P(mix|n)=1-P(res|n)-P(susc|n)-P(miss|n) \end{matrix}$$

The above formulae also hold for $n=0$, since e.g.

$$P(susc|0)=\left((1-p)+p(1-q)\right)^{0}-(1-q)^{0}=1-1=0$$

Summing over all possible values of *n*

$$P(outcome)=\sum_{n=0}^{\infty} P(n)P(outcome|n)$$

For a discrete random variable $N$ on the non-negative integers such as the MOI, the probability generating function is defined for real numbers $z$ by

$$G(z)=\sum_{n=0}^{\infty} P(N=n)z^{n}$$

So we can write the probabilities of each outcome in terms of $G$

$$\begin{matrix} P(res)=\sum_{n=0}^{\infty} P(n)P(res|n) \\ =\sum_{n=0}^{\infty} P(n)\left[\left(p+(1-p)(1-q)\right)^{n}-(1-q)^{n}\right] \\ =G(p+(1-p)(1-q))-G(1-q) \\ P(susc)=G((1-p)+p(1-q))-G(1-q) \\ P(miss)=G(1-q) \\ P(mix)=1-P(res)-P(susc)-P(miss) \end{matrix}$$

If we assume that the MOI are Poisson distributed with mean $\mu$, then

$$G(z)=e^{\mu(z-1)}$$

and so

$$\begin{matrix} P(res)=e^{\mu\left(p+(1-p)(1-q)-1\right)}-e^{-\mu q} \\ P(susc)=e^{\mu\left((1-p)+p(1-q)-1\right)}-e^{-\mu q} \\ P(miss)=e^{-\mu q} \end{matrix}$$

If instead $n$ has a negative binomial distribution with mean $\mu$ and dispersion parameter $r$, the probability generating function is

$G(z)=\left(\frac{1-p_{NB}}{1-p_{NB}z}\right)^{r}$, where $p_{NB}=\frac{\mu}{\mu+r}$

So in terms of $\mu$ and $r$

$$G(z)=\left(\frac{r}{\mu+r-\mu z}\right)^{r}$$

$$\begin{matrix} P(res)=G(p+(1-p)(1-q))-G(1-q) \\ =r^{r}\left[\frac{1}{\left(\mu+r-\mu(p+(1-p)(1-q))\right)^{r}}-\frac{1}{\left(\mu+r-\mu(1-q)\right)^{r}}\right] \end{matrix}$$

$$P(susc)=r^{r}\left[\frac{1}{\left(\mu+r-\mu((1-p)+p(1-q))\right)^{r}}-\frac{1}{\left(\mu+r-\mu(1-q)\right)^{r}}\right]$$

$$P(miss)=\frac{r^{r}}{\left(\mu+r-\mu(1-q)\right)^{r}}$$

### Likelihood contributions

The preceding probabilities can be used to write the likelihood contributions of each individual. Since we only observe samples which are not all missing, we need a truncated likelihood, dividing by the probability that the sample is not all missing.

If the MOI $n$ is known, then the likelihood for each outcome (res, susc or mix) is given by

$$L(n,outcome)=\frac{P(n)P(outcome|n)}{1-P(miss)}$$

using the formulae in equations - in the numerator and the formula for $P(miss)$ from equations .

If the MOI is not known, then the likelihood uses the formulae in equations

$$L(outcome)=\frac{P(outcome)}{1-P(miss)}$$

The overall likelihood is the product of each individual’s likelihood contribution, and both types of likelihood may be used if the MOI is only known for some people in the dataset.

### Extensions to the model

We tried 3 further extensions to the model in order to vary assumptions about the detectability of clone and to try to better fit the proportion of mixed infections in the data. These models were all fitted to the full individual level test dataset from Tanzania described in the main text, estimating frequencies for each cluster and year.

1. assuming that clones may be undetected in an infection with any MOI, but that the probability of missing a clone increases linearly with MOI.

The calculations are as above but instead of being a constant, *q* depends on MOI and is given by:

$$q=q_{0}+q_{1}n$$

We estimated $q_{0}$, $q_{1}$and the frequency simultaneously. Maximum likelihood estimates were $q_{0}=0.65$ and $q_{1}=-0.025$ across all clusters and years.

(2) assuming that in each multiclonal infection, there is a single dominant clone that is detected with probability $q_{1}$, and all other clones are detected with probability $q_{2}$, where we expect $q_{1}\geq q_{2}$. If there are $n$ clones in total and $c$ resistant clones, then the dominant clone is resistant with probability $c/n$.

Now we have for $n>0$

$$P(res|n,c)=\left(\frac{n-c}{n}\right)(1-q_{1})(1-q_{2})^{n-c-1}+\frac{c}{n}(1-q_{2})^{n-c}-(1-q_{1})(1-q_{2})^{n-1}$$

Summing over all possible values of $c$

$$P(res|n)=\left(p+(1-p)(1-q_{1})\right)\left(p+(1-p)(1-q_{2})\right)^{n-1}-(1-q_{1})(1-q_{2})^{n-1}$$

$$P(susc|n)=\left((1-p)+p(1-q_{1})\right)\left((1-p)+p(1-q_{2})\right)^{n-1}-(1-q_{1})(1-q_{2})^{n-1}$$

$$P(miss|n)=(1-q_{1})(1-q_{2})^{n-1}$$

For $n=0$, $P(miss|0)=1$, $P(res|0)=0$, $P(susc|0)=0$.

Summing over all values of $n$

$$\begin{matrix} P(miss)=\sum_{n=0}^{\infty} P(n)P(miss|n) \\ =\sum_{n=0}^{\infty} P(n)(1-q_{1})(1-q_{2})^{n-1}+P(0)-P(0)(1-q_{1})(1-q_{2})^{-1} \\ =\left(\frac{1-q_{1}}{1-q_{2}}\right)G(1-q_{2})+\left(\frac{q_{1}-q_{2}}{1-q_{2}}\right)P(0) \end{matrix}$$

where $G(.)$ is the probability generating function for the distribution of $n$.

Similarly,

$$P(res)=\left(\frac{p+(1-p)(1-q_{1})}{p+(1-p)(1-q_{2})}\right)G\left(p+(1-p)(1-q_{2})\right)+\left(\frac{p(q_{1}-q_{2})}{p+(1-p)(1-q_{2})}\right)P(0)-P(miss)$$

$$P(susc)=\left(\frac{(1-p)+p(1-q_{1})}{(1-p)+p(1-q_{2})}\right)G\left((1-p)+p(1-q_{2})\right)+\left(\frac{(1-p)(q_{1}-q_{2})}{(1-p)+p(1-q_{2})}\right)P(0)-P(miss)$$

For the Poisson model, where $G(z)=e^{\mu(z-1)}$ and $P(0)=e^{-\mu}$, we have

$$P(miss)=\left(\frac{1-q_{1}}{1-q_{2}}\right)e^{-\mu q_{2}}+\left(\frac{q_{1}-q_{2}}{1-q_{2}}\right)e^{-\mu}$$

$$\begin{matrix} P(res)=\left(\frac{p+(1-p)(1-q_{1})}{p+(1-p)(1-q_{2})}\right)e^{\mu\left(p+(1-p)(1-q_{2})-1\right)} \\ +\left(\frac{p(q_{1}-q_{2})}{p+(1-p)(1-q_{2})}\right)e^{-\mu}-P(miss) \end{matrix}$$

$$\begin{matrix} P(susc)=\left(\frac{(1-p)+p(1-q_{1})}{(1-p)+p(1-q_{2})}\right)e^{\mu\left((1-p)+p(1-q_{2})-1\right)} \\ +\left(\frac{(1-p)(q_{1}-q_{2})}{(1-p)+p(1-q_{2})}\right)e^{-\mu}-P(miss) \end{matrix}$$

Then the truncated likelihood can be found as before. $G(z)$ for the negative binomial model could be used instead.

Fitting to the full individual test dataset, our best estimates of the parameters were $q_{1}=0.93$ and $q_{2}=0.41$.

(3) assuming there is geographical heterogeneity in the proportion of resistant clones.

Mixed infections could be under-represented due to such geographical heterogeneity. At the extreme, if half the population was from an area with no resistance and half from an area with all resistance, then there would be no mixed clones.

Suppose that $p$ varies in the population according to a Beta distribution with parameters $\alpha=\theta p_{0}$ and $\beta=\theta(1-p_{0})$, so that there is a mean of $p_{0}$ and small values of $\theta$ mean more heterogeneity. Let $f$ be this Beta probability density function. Now we have

$$P(res)=\int_{0}^{1} P(res|p)f(p) dp$$

For the Poisson case,

$$\begin{matrix} P(res|p)=e^{\mu\left(p+(1-p)(1-q)-1\right)}-e^{-\mu q} \\ =e^{-\mu q}\left(e^{\mu qp}-1\right) \end{matrix}$$

Hence

$$P(res)=e^{-\mu q}\left(\psi(\mu q)-1\right)$$

$\psi(.)$ is the moment-generating function of the beta distribution,

$$\psi(x)={}_{1}F_{1}\left(\alpha,\alpha+\beta,x\right)$$

where ${}_{1}F_{1}(.)$ is the confluent hypergeometric function ^1^.

Hence

$$P(res)=e^{-\mu q}\left({}_{1}F_{1}\left(\theta p_{0},\theta,\mu q\right)-1\right)$$

$$P(susc)=e^{-\mu q}\left({}_{1}F_{1}\left(\theta(1-p_{0}),\theta,\mu q\right)-1\right)$$

$P(miss)=e^{-\mu q}$, as before.

${}_{1}F_{1}(.)$ can be calculated in R using the library gsl and the function hyperg_1F1.

For the negative binomial model, we can use the result that

$\int_{0}^{1} \frac{x^{\alpha-1}(1-x)^{\beta-1}}{(1-ax)^{\gamma}} dx=B(\alpha,\beta) _{2}F_{1}(\gamma,\alpha,\alpha+\beta;a)$ for $|a|<1, \alpha,\beta>0$

where $B(.)$ is the beta function and $_{2}F_{1}(.)$ is the hypergeometric function ^2^.

$$\begin{matrix} P(res|p)=\frac{r^{r}}{\left(\mu+r-\mu(p+(1-p)(1-q))\right)^{r}}-P(miss|p) \\ =\left(\frac{r}{r+\mu q}\right)^{r}\frac{1}{\left(1-\mu qp/(r+\mu q)\right)^{r}}-P(miss|p) \end{matrix}$$

$P(res)=\int_{0}^{1} P(res|p)f(p) dp$, with $f(p)=\frac{p^{\theta p_{0}-1}(1-p)^{\theta(1-p_{0})-1}}{B(\theta p_{0},\theta(1-p_{0}))}$

So

$$\begin{matrix} P(res)=\left(\frac{r}{r+\mu q}\right)^{r}{}_{2}F_{1}\left(r,\theta p_{0},\theta;\mu q/(r+\mu q)\right)-P(miss) \\ P(susc)=\left(\frac{r}{r+\mu q}\right)^{r}{}_{2}F_{1}\left(r,\theta(1-p_{0}),\theta;\mu q/(r+\mu q)\right)-P(miss) \end{matrix}$$

With heterogeneity in $p$, where the MOI $n$ is known, we have

$$P(miss|n)=(1-q)^{n}$$

$$\begin{matrix} P(susc|n,p)=\left((1-p)+p(1-q)\right)^{n}-(1-q)^{n} \\ =\left(1-pq\right)^{n}-(1-q)^{n} \end{matrix}$$

$$\begin{matrix} P(susc|n)=\int_{0}^{1} [\left(1-qp\right)^{n}-(1-q)^{n}]f(p) dp \\ =\int_{0}^{1} \left(1-qp\right)^{n}f(p) dp-(1-q)^{n} \\ =_{2}F_{1}(-n,\theta p_{0},\theta;q)-(1-q)^{n} \end{matrix}$$

$$P(res|n)=_{2}F_{1}(-n,\theta(1-p_{0}),\theta;q)-(1-q)^{n}$$

This could be combined with the Poisson or negative binomial models, since the probabilities are conditional upon $n$.

Fitting to the full individual test dataset, our best estimates of the parameters were $q=0.711$ and $\theta=3.1$.

Supplementary Table 1. Maximum log likelihood fits of models to the prevalence of pure resistant, pure wild type and mixed infections in the 24 clusters in 2 years in the full individual level test dataset from Tanzania. Smaller negative log likelihood indicates a better fit.

| Model & assumptions | Negative Log likelihood |
| --- | --- |
| 100% detection of clones: equations 2 and 3 in the main text (Method 2A) | 2109.59 |
| Detection is imperfect only in higher MOI infections: equation 4 in main text, genotyping sensitivity limit fixed at 33% (Method 2B) | 1772.48 |
| Constant probability of detecting a clone independent of MOI: equation 6 in the main text. (Method 2C) | 1616.23 |
| Probability of detecting each clone declines with MOI (extended Method 2: see supplement above) | 1611.06 |
| Geographical heterogeneity in p; constant probability of detecting a clone independent of MOI: (extended Method 2: see supplement above). | 1610.64 |
| In multiclonal infections, there is a single dominant clone that is detected with higher probability than all other clones (extended Method 2: see supplement above) | 1610.55 |

R code for estimating mutation frequencies in the parasite population from the prevalence of mutations in humans, when MOI data are not available but the proportion of mixed wild type-resistant infections is known. The following code uses method 5C defined in this paper, assuming Poisson-distributed MOI (equation 8 in Methods). Code for other methods described in this paper is available upon request to the corresponding author.

### define data

n_pos_r <- 20 # number of people in whom resistant parasites observed

n_pos_wt<- 50 # number of people in whom wild type parasites observed

n<-50 # total number of people (may be <n_pos_r+n_pos_wt due to mixed infections)

### define parameters

# q = probability that a resistant or wild type clone is detected in the data

# (can change this to 1 to assume 100% detection)

q<- 0.54

# to estimate mean MOI using slide-prevalence of malaria infection:

# if slide-prevalence is unknown, first obtain estimates of slide prevalence in 2-10 yr olds

# at the location of the survey, using the malaria atlas project maps and raster files

# http://www.map.ox.ac.uk/

slide_2_10<-0.38 # enter proportion slide-positive in 2-10 yr olds (0-1 scale)

# now estimate slide prevalence in 0-15 yr olds

# read in supplementary dataset 3 file: slide_convert_2_10_0_15.csv

slide_convert<-read.csv("slide_convert_2_10_0_15.csv")

diff<-abs(slide_convert$slide_pos_2_10 - slide_2_10)

slide_0_15<-slide_convert$slide_pos_0_15[which(diff==min(diff))]

a<- 1.08174

b<- 0.18504

logit_slide_0_15 <- log(slide_0_15/(1-slide_0_15))

mean_moi<- exp(a + b*logit_slide_0_15) # mean population MOI

# mean_moi<- 3.0 # alternatively, simply enter the mean MOI if known

### calculate the expected proportion of all-resistant, all-wild type and mixed

# resistant-wild type infections given a frequency, mean MOI and q.

prev.r.mix.wt <-function(freq,mean_moi,q) {

pdet_none <- exp(-mean_moi*q)

pdet_pure_r<- exp(mean_moi*(freq+(1-freq)*(1-q)-1)) - pdet_none

pdet_pure_wt <- exp(mean_moi*((1-freq)+freq*(1-q)-1)) - pdet_none

pdet_mix <- 1- pdet_pure_r - pdet_pure_wt - pdet_none

resultr<- pdet_pure_r / (1-pdet_none)

resultwt<- pdet_pure_wt / (1-pdet_none)

resultmix<- pdet_mix / (1-pdet_none)

return(c(resultr,resultmix,resultwt))

}

## function to calculate the log likelihood given a particular frequency

calc_loglik<- function(freq, mean_moi, n_pos_r, n_pos_wt, n, q) {

prevs<-prev.r.mix.wt(freq,mean_moi,q)

n_mix<-n_pos_r+n_pos_wt-n # calculate number mixed

n_pure_r<-n_pos_r-n_mix # calculate number with all-resistant infections

n_pure_wt<-n_pos_wt-n_mix # calculate number with all-wild type infections

loglik<- n_pure_r*log(prevs[1]) + n_mix*log(prevs[2]) + n_pure_wt*log(prevs[3])

return(loglik)

}

## find the frequency which maximises the likelihood, using the R optimise function

temp<-optimize(f=calc_loglik, interval=c(0.00001, 0.9999), mean_moi=mean_moi, n_pos_r=n_pos_r, n_pos_wt=n_pos_wt, n=n,q=q, maximum = TRUE)

est.freq<-temp$maximum ## the estimated frequency of resistance in the survey.

Supplementary Figure S1. Predicted and observed (A) slide prevalence (PfPR) in 0-15 year olds and (B) population mean MOI in 24 clusters in 2004 and 2007 the test dataset in Tanzania. Slide-prevalence estimates in 2-10 year olds were taken from the Malaria Atlas Project and age-adjusted to 0-15 year olds using a published model.^3^ Mean MOI was estimated from the relationship in Figure 4 in the main text.


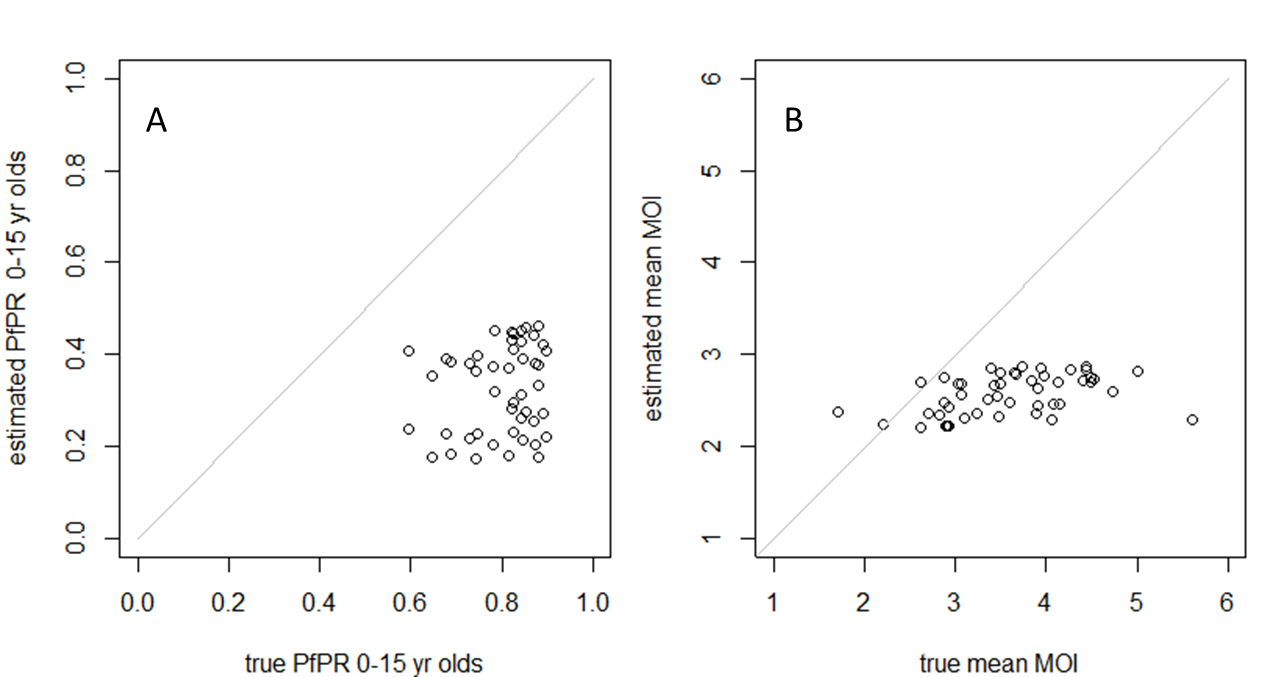


Supplementary Figure S2. Predicted and observed proportion of mixed wild type and resistant infections in the test dataset under different models. Observed proportions of mixed infections are from cross-sectional surveys assessing *K540E* in 2004 and 2007 in 24 divisions in Tanzania. The predicted proportion of mixed infections depends on the frequency of resistance and the assumptions about detection of clones in different models. Frequencies were estimated from data on mixed infections and mean MOI was estimated using the Malaria Atlas Project slide prevalence and the relationship in Figure 4. We assumed either 100% detection of clones (black); that clones are missed in high MOI infections (red; equation 4 in Methods) or that clones have a constant probability of being missed in any infection (blue; equation 8 in Methods). Compared to the data, these predictions had mean squared errors of 0.030, 0.014 and 0.020, respectively, indicating that imperfect detection of clones substantially improved the fit.
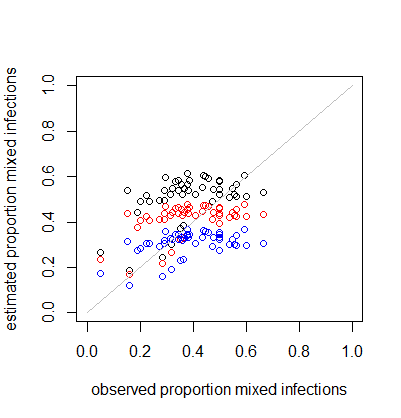


Supplementary Figure S3. Predicted and observed proportion of mixed wild type and resistant infections in the systematic review 540E data under different models. Frequencies were estimated from data on mixed infections and mean MOI was estimated using the Malaria Atlas Project slide prevalence and the relationship in Figure 4 in the main text. We assumed either 100% detection of clones (purple); that clones are missed in high MOI infections (blue; equation 4 in Methods) or that clones have a constant probability of detection in any infection equation 8 in Methods (orange q=0.54; red q=0.30). Compared to the data, these predictions had mean squared errors of 0.018, 0.014 and 0.012 and 0.018, respectively.


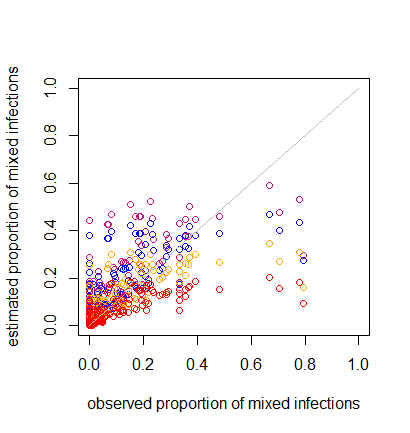


Supplementary Figure S4. Spatiotemporal variation in 540E and 581G frequency. (A) Different spatial scales. We compared prevalence of the same mutation in the same country between surveys done within 1 year of each other. We plot the pairwise distance between the surveys against the absolute difference in prevalence of the mutation. Blue squares indicate the proportion of survey pairs in which the difference in mutation prevalence was less than 10%. Temporal trends in (B) 540E and (C) 581G frequency. We compared successive measures within the same first administrative area which were <100km apart. Comparisons of two zero prevalence measures are excluded in all panels.


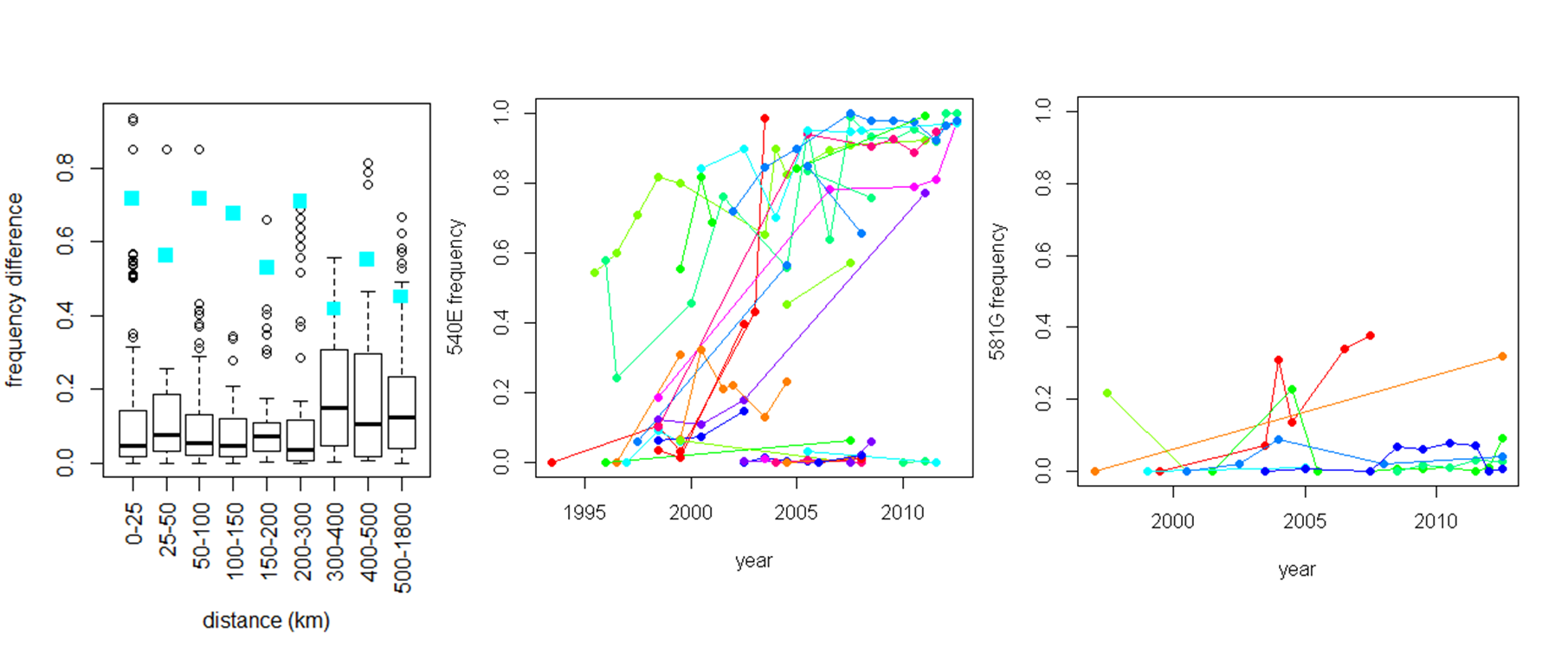


References

1. H. B. Tables of integral transforms. California Institute of Technology Bateman Manuscript Project, New York: McGraw-Hill, edited by Erdelyi, Arthur 1954; 1. 1954.

2. Whittaker ET WG. A course of modern analysis: Cambridge university press;. 1996.

3. Griffin JT, Ferguson NM, Ghani AC. Estimates of the changing age-burden of Plasmodium falciparum malaria disease in sub-Saharan Africa. *Nat Commun* 2014; **5**: 3136.
